# RET Protein Expression in Colorectal Cancer; An Immunohistochemical Assessment

**DOI:** 10.31557/APJCP.2021.22.4.1019

**Published:** 2021-04

**Authors:** Maryam Ashkboos, Mehdi Nikbakht, Giti Zarinfard, Mitra Soleimani

**Affiliations:** *Department of Anatomical Sciences, Isfahan University of Medical Sciences, Isfahan, Iran. *

**Keywords:** CRC, MBD1, clinicopathological features, ICH

## Abstract

**Background:**

RET (rearranged during transfection) is a transmembrane receptor tyrosine kinase and a receptor for the GDNF-family ligands. It plays the role of a tumor suppressor in colorectal cancer. Therefore, it is expected that RET gene becomes downregulated in colorectal cancer (CRC). In this study, we evaluated immuno-histochemical expression of *RET* in *CRC* and assessed its correlation with some of the clinicopathological features to study the prognostic value in CRC.

**Materials and Methods:**

In total, 60 cases of colorectal cancer (CRC) from the patients who underwent surgical gastroenterology operations were randomly selected. The samples included one tumor-rich section per case and one adjacent tumor-free section as the normal control for that case. Then, immunohistochemistry (ICH) was performed for RET on all the samples and the expression of *RET* was analyzed. Furthermore, the correlation of RET with clinicopathological features including age, gender, location of the tumor, grade, and stage was evaluated.

**Results:**

The expression of *RET* caused significant downregulation in cancer samples compared to the normal control ones (P = 0.002). This downregulation increased in correlation to both grade and metastasis to lymph nodes (P = 0.03 & 0.02 respectively). However, no correlation was found between the expression of *RET* and gender as well as location of the tumor.

**Conclusion:**

RET may be considered as a protein marker in CRC detection and prognosis.

## Introduction

Colorectal cancer (CRC) is the third leading cause of cancer-related death worldwide. One in twenty has the risk of developing CRC during their lifetime (Bardhan et al., 2013). CRC is characterized by certain genetic and epigenetic changes that induce proliferative activity and inhibit apoptosis (Fennell et al., 2018). Activating proto-oncogenes or de-activating some tumor suppressor genes can lead to cancer (Munteanu et al., 2015). DNA hypermethylation is frequently associated with transcriptional silencing of tumor suppressor genes (Ng and Yu, 2015). Several tumor suppressor genes have been already introduced that are involved in the CRC pathogenesis. Among the most widely known genes are *APC, p 53, p27, MSI, LOH 18q, deletion 5 q allele*, and DNA hypermethylation (Munteanu et al., 2015). DNA hypermethylation is a type of aberrant epigenetic alteration. CRC alterations were first identified in 1980s (Armaghany et al., 2015). A subtype of CRC, i.e. CIMP (CpG island methylator phenotype) is characterized as an epigenome fraught with methylated genes. CIMP CRCs are associated with older age, female gender, family history of CRC, proximal location in the colon, and mucinous cell differentiation (Weisenberger et al., 2006). Recently, it is found that RET (rearranged during transfection) plays a tumor suppressor role in CRC (Luo et al., 2013). RET is a transmembrane receptor tyrosine kinase and a receptor for the GDNF-family ligands. It was one of the first oncogenes that was identified. It has long been reported that specific mutations in RET are associated with medullary thyroid carcinoma (Mulligan et al., 1994). The association between RET and hirschsprung’s disease (developmental disorder of the enteric nervous system) is well-recognized as its major responsible gene (Tomuschat and Puri, 2015). Recently, the tumor suppressor functions of RET have been taken into consideration. It has been shown that RET is a conditional tumor suppressor gene in the CRC through its dual role: mutation and aberrant methylation (Grady, 2013). In a recent study, it was concluded that RET is the second of nine genes that exclusively hypermethylate in CIMP CRC (Wei et al., 2016). Considering the tumor suppressor functions of RET in CRC and since in *CIMP, RET* expression is correlated to clinicopathologic features, in this study, we aim to assess the *RET* expression in cancer and normal tissues and to evaluate the correlation of *RET* expression with clinicopathological features.

## Materials and Methods

*Case Selection and Tissue Samples*

In total, 60 cases of colorectal cancer (CRC) and 60 cases of normal adjacent tissues (more than 10 cm far from the margin of tumors) were collected as paraffin embedded blocks prepared at Alzahra Hospital (Isfahan, Iran) and assessed in a longitudinal retrospective study. All the patients sustained either elective or emergency surgical operations. The non-resected tumors or those with antineoplastic therapies were excluded from the study. The frequency and distribution of clinicopathologic features are depicted in Table 1.

*Immunohistochemistry (ICH)*

Immunohistochemical analysis of RET was performed as previously described protocol (Yemelyanova et al., 2011). Briefly, the sections with 5 µm thickness from formalin-fixed paraffin-embedded blocks were deparaffinized in xylene and rehydrated in a graded series of ethanol. Next, the sections pretreated with 0.01 mol/L citrate buffered saline (pH 6.0) and autoclaved at 121°C for 15 min for antigen retrieval. Endogenous peroxidase activity was blocked by incubation with 3% H_2_O_2_ for 30 min at the room temperature. Nonspecific binding of the immunological reagents was blocked by incubating the sections with 10% normal goat serum for 1 h. Then, the sections were incubated with rabbit antibody against human RET (#ab134100, Abcam, UK) at 4°C overnight and (after washing) incubated with anti-rabbit secondary antibody (#ab205718) for 30 min RT. The peroxidase activity was developed with 3,3′-diaminobenzidine etrahydrochloride (DAB) in the sterile H_2_O_2_ solution for 5 min. Nuclear counterstaining was done using Mayer’s hematoxylin solution. Two blinded observers (Sh.G and ZA) independently examined the immunostained sections. 

*Cell counts and scoring system*

**Figure 1:**
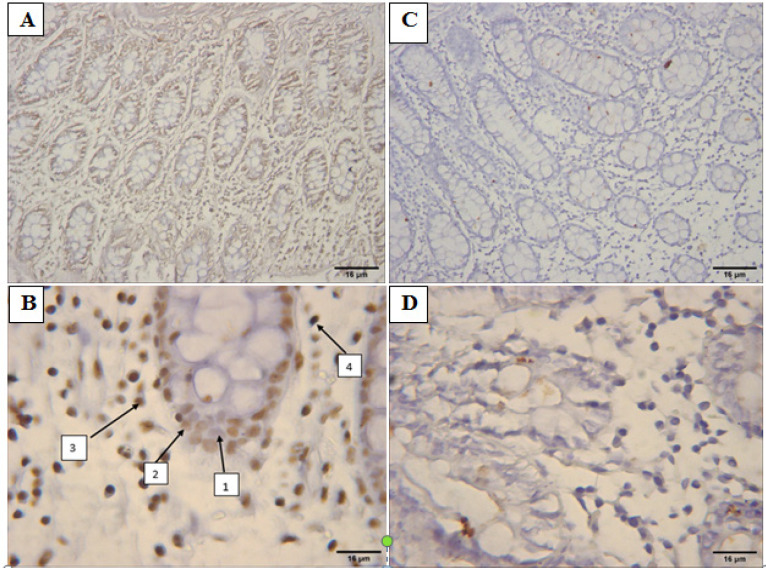
A & B, The Expression of *RET* in Normal Sections Compared to C & D, CRC(Non-Mucinous) Stained Samples (100 & 400X Magnification). Brown stained cells are considered as positive cells for *RET*. B) Different intensity of positive stained cells for *RET *(1=non-stained, 2= mild, 3=moderate, 4=sever)

**Figure 2:**
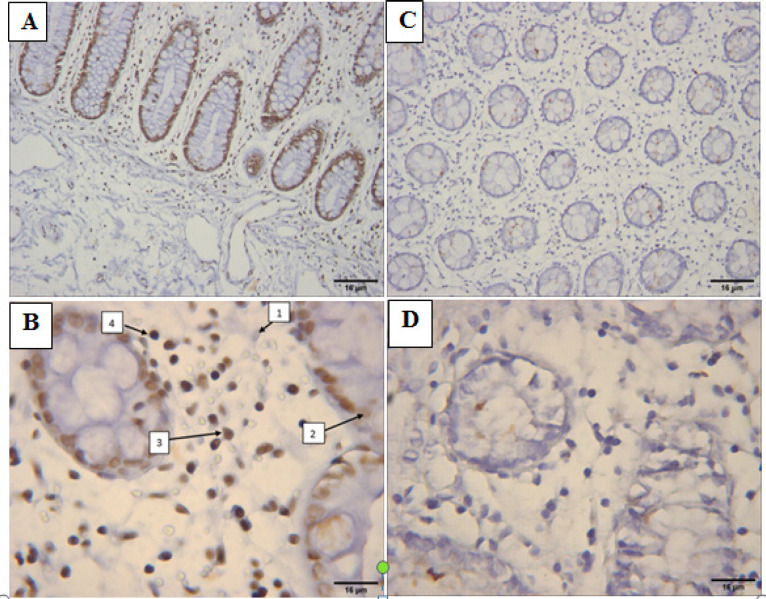
A & B, The Expression of *RET* in Normal Sections Compared to C & D, CRC (Mucinous) Stained Samples (100 & 400X Magnification). Brown stained cells are considered as positive cells for *RET*. B) Different intensity of positive stained cells for *RET* (1=non-stained, 2= mild, 3=moderate, 4=sever)

Stained sections were observed by the light microscope and at least 500 cells in 10 different fields were picturized by Motic Image Advanced Plus 3. Cell count was done in ImageJ 1.52n software. The staining intensity was assessed on a four-point scale as follows: 0= Negative, 1= Weak, 2= Intermediate, and 3= Strong (Figures 1B and 2B). Total intensity per section was calculated by the following equation: H-score = Ʃ(1+i) pi.

*Statistical analysis*

Statistical analysis was performed in SPSS software (v. 25) using paired t-student test, independent t-test, Pearson’s correlation coefficient, and Spearman’s rank correlation coefficient to compare protein expression difference between the tumor and normal adjacent samples and to evaluate the correlation of *RET* expression with the features of gender, age, location, type, grade, and stage of tumor. Data were represented as mean ± SD and p-values of less than 0.05 were considered as statistically significant. P < 0.05 was considered significant.

**Table 1:** The Mean Expression of *RET* Protein in Cancer Samples Compared to Normal Adjacent Samples

	Mean	SD	P-value
Cancer	50.9	7.8	<0.002
Normal	107.01	14.7	

**Table 2:** The Mean Expression of RET Protein in Cancer Samples Compared to Normal Adjacent Samples

	Male		Female		P-value
	Mean	SD	Mean	SD	
Cancer	47.5	12.6	53.1	9.9	0.21
Normal	140.4	25.1	89.03	17.7	0.046

**Figure 3:**
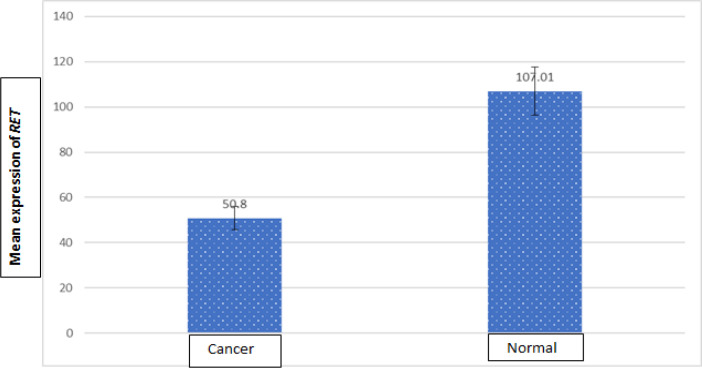
The Mean Expression of RET Protein in Cancer Samples Compared to Normal Samples

**Table 3:** The Expression of RET Protein Expression Correlation to Gender

	Age	
	r	P-value
Cancer	- 0.031	0.81
Normal	- 0.275	0.03

**Table 4:** The Pearson Correlation Coefficient of RET Protein Expression Correlation to Age

Tumor Type	Mean	SD	P-value
Mucinous	53.1	8.5	0.53
Non-mucinous	39.8	20.1	

**Table 5:** The Mean Expression of RET Protein in Cancer Samples Correlation to Tumor Type

Tumor Location	Mean	SD	P-value
Colon	48.4	9.8	0.64
Rectosigmoid	56.3	13.2	

**Figure 4:**
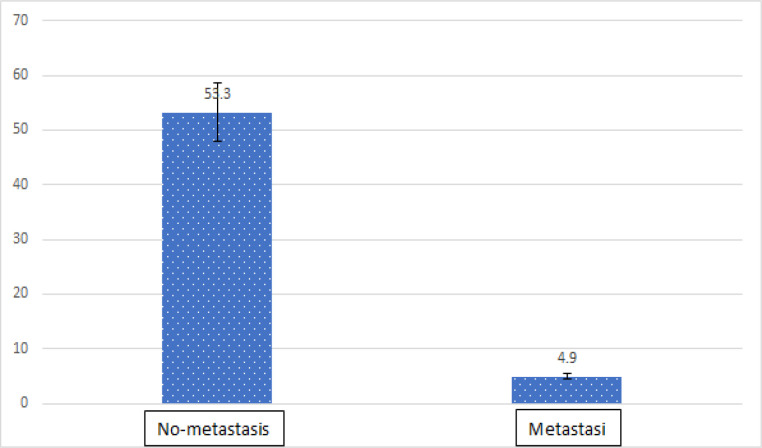
The Mean Expression of RET Protein in Cancer Samples Compared to Normal Samples Correlation to Tumor Type

**Table 6:** The Mean Expression of RET Protein Correlation to Tumor Location

Lymph node metastasis	Mean	SD	P-value
Yes	53.3	8.1	0.02
No	4.9	1.7	

**Table 7:** The Mean Expression of RET Protein in Cancer Samples Compared to Normal Samples Correlation to Tumor Type

	RET Expression	
	r	P-value
Stage	- 0.157	0.28
Grade	- 0.248	0.03

## Results

*General observation of samples*

The cells with brown stained cytoplasm for RET with various staining in non-mucinous (Figures 1C and D) and mucinous (Figures 2 C and D) cancer sections and normal sections (Figures 1 (A and B) and Figures 2 (A and B) were observed. Significant reduction in RET protein expression was noted in the tumor samples in comparison to the normal control samples. 

*Correlation of RET expression and clinicopathologic features*

The statistical analysis of the data showed a significant difference in the expression of *RET* in cancer samples in comparison to the normal adjacent samples (P<0.002). No significant correlation was found between the expression of *RET* and gender (P=0.21). However, the expression of *RET* was significantly higher in the normal female samples compared to the normal male samples (P=0.046). Pearson’s correlation coefficient showed no significant correlation between *RET* expression and age (P=0.54). Although a conversely significant correlation was seen between *RET* expression and age in the normal samples (P=0.03), there was no significant correlation between *RET* expression in cancer samples compared to the normal samples in terms of the type and location of the tumors (P= 0.53 and 0.64, respectively). Moreover, a significant difference was found in *RET* expression between stages (P=0.28). However, *RET* expression showed a significant difference in the samples with metastasis to lymph nodes from the samples without metastasis to lymph nodes (0.02). Spearman’s rank correlation coefficient showed a significant difference in *RET* expression in terms of the grade of tumors (P=0.03). The statistics is described in Tables 3-7 and Figures 3-4.

## Discussion

In this study, we assessed the expression of *RET* in colorectal cancer and evaluated the correlation of *RET *expression to clinicopathologic features.* RET* gene is one of the first oncogenes that has been identified (Mulligan et al., 1994). Its role as a proto-oncogene has been known for many years (Nakamura et al., 1994). However, its account as a tumor suppressor gene recently is taken into consideration. It has been recently reported that RET is methylated in 63% of colorectal cancers and that, in 27% of colon adenomas, RET is in the methylated format. Therefore, tumor suppressor activity of RET in the colon cancer is suggested (Luo et al., 2013). In the integrated analysis of genome-wide DNA methylation and gene expression profiling study, *RET* was the second of nine potential biomarkers that was introduced for the rectal cancer (Wei et al., 2016). In our study, we found* RET* expression was significantly lower in CRC than in the normal samples. This was in concordance with previous studies, demonstrating the significant downregulation of RET during colon and rectal cancers. The data of this study showed that the expression of *RET* was in correlation with grade and metastasis to lymph nodes. However, no correlation was found between *RET* expression and gender, age, location, and stage of the tumors. 

In conclusion, the results from this study suggested that, firstly, *RET* is involved in the pathogenesis of colorectal cancer. Secondly, it is in correlation with the tumor grade and metastasis. However, to discuss RET consideration as a prognosis factor with more certainty, a larger sample size than the one used in this study is required. 

## Author Contribution Statement

The authors confirm contribution to the paper as follows: study conception and design: Mitra Soleimani, Maryam Ashkboos, Mehdi Nikbakht; data collection: Mehdi Nikbakht, Maryam Ashkboos; analysis and interpretation of results: Mitra Soleimani, Maryam Ashkboos, Giti Zarinfard; draft manuscript preparation: Mitra Soleimani. All authors reviewed the results and approved the final version of the manuscript.
